# A weighted multi-scale attention-enhanced temporal convolutional network for motor imagery EEG decoding in brain-computer interfaces

**DOI:** 10.3389/fbioe.2026.1842610

**Published:** 2026-06-03

**Authors:** Zhongchen Song, Xuejun Zhang

**Affiliations:** 1 School of Electronic and Optical Engineering & Flexible Electronics (Future Technology), Nanjing University of Posts and Telecommunications, Nanjing, China; 2 Nation-Local Joint Project Engineering Lab of RF Integration & Micropackage, Nanjing University of Posts and Telecommunications, Nanjing, China

**Keywords:** attention mechanism, brain-computer interface, channel-preserving prior path, electroencephalogram, motor imagery, temporal convolutional network, weighted feature fusion

## Abstract

Accurate decoding of motor imagery electroencephalogram signals plays a critical role in brain-computer interfaces for neurorehabilitation and assistive technologies. However, existing multi-scale temporal methods often overlook scale-specific importance and fail to jointly capture transient and long-term neural dynamics, we propose a Weighted Multi-scale Attention-enhanced Temporal Convolutional Network (WMA-TCNet). The model employs parallel multi-scale temporal convolutions to capture neural patterns associated with distinct EEG rhythms. A global-aware scale attention mechanism adaptively weights each branch to emphasize task-relevant temporal information. A weighted Channel-Preserving Prior Path is introduced to maintain channel-wise dependencies and enhance spatial modeling stability across cortical regions. In addition, a temporal attention-guided TCN jointly captures local and long-range temporal dependencies. Experiments on BCI Competition IV 2a and 2b datasets show that WMA-TCNet achieves accuracies of 85.8% and 90.0% in subject-dependent settings, and 68.6% and 79.5% in cross-subject scenarios. These results demonstrate improved decoding performance and robustness, while providing a biologically meaningful framework for modeling multi-scale neural dynamics, with potential applications in brain-computer interfaces and neurorehabilitation.

## 1 Introduction

Brain-computer interface (BCI) technology, which serves as a bridge between the brain and external devices, has achieved significant progress in fields such as medicine, rehabilitation, and intelligent control systems (Al-Saegh et al., 2021).Motor imagery (MI) has attracted considerable attention in recent years as an important BCI paradigm. MI refers to a mental process in which the brain imagines a motor action without actual execution by the muscles or limbs, and it can be used to infer the user’s motor intention (Musallam et al., 2021).

In MI tasks, decoding electroencephalogram (EEG) signals associated with MI enables researchers to provide effective assistive tools for stroke patients (Nanthini et al., 2022), individuals with disabilities (Zhang et al., 2023), and others who require alternative control systems. MI-EEG signals are generated during imagined movements through the activation of motor-related brain regions and exhibit distinct spatiotemporal patterns. These patterns are characterized by event-related desynchronization/synchronization (ERD/ERS) in the μ (8–13 Hz) and β (13–30 Hz) rhythms (Miao et al., 2026).

However, during signal decoding, artifacts such as facial muscle activity, eye blinks, and environmental electromagnetic interference may contaminate MI-EEG signals, leading to reduced decoding accuracy (Tang et al., 2023). In addition, the decoding performance of MI-EEG signals can be influenced by variations in brain structure and function across subjects. These differences arise from individual physiological characteristics, cognitive abilities, neural adaptability, and training backgrounds (Wan et al., 2021). Therefore, effectively identifying the true neural intention from complex EEG signals remains a major challenge for the widespread application of BCIs.

Traditional machine learning (ML) methods typically employ fixed filter banks, such as filter bank common spatial pattern and its variants (Qamhan et al., 2021), to decompose EEG signals into multiple frequency bands and apply spatial filters. However, these approaches rely on manually predefined frequency bands and filters, which limits their adaptability to individual differences and ultimately affects classification performance. Moreover, the effectiveness of ML methods depends heavily on expert knowledge. For example, although independent component analysis can separate artifacts (Craik et al., 2019), improper removal may lead to the loss of critical MI-related information.

In recent years, deep learning has been widely applied to signal decoding tasks, providing an end-to-end capability to automatically learn features from MI-EEG data. In MI classification tasks, previous studies have integrated various deep learning architectures, including convolutional neural networks (CNN) (Varone et al., 2024), recurrent neural networks (RNN) (Mirsalari et al., 2020), and temporal convolutional networks (TCN) (Ingolfsson et al., 2020). Among these, CNNs have been extensively used for decoding MI-EEG signals, leading to the development of a variety of CNN-based architectures (Ali et al., 2024).

In this context, some researchers have focused on designing lightweight CNN architectures. For example, Sun and Kintak (2022) proposed a lightweight residual convolutional network that can directly process raw EEG data. Sun et al. (2021) further introduced a model based on sparse spectral-temporal decomposition, squeeze-and-excitation blocks, and convolutional neural networks, achieving superior performance in terms of accuracy, efficiency, and robustness. Cao et al. (2024) proposed a lightweight end-to-end three-domain feature fusion network that balances model size and decoding performance. However, single-scale CNNs mainly focus on local features and often struggle to effectively capture the temporal characteristics of different rhythmic components in MI-EEG signals. End-to-end lightweight CNNs still show limitations when dealing with inter-subject variability and the non-stationary nature of EEG signals. To address this issue, some studies have introduced multi-branch architectures. Jia et al. (2021) designed a multi-branch multi-scale CNN that captures feature differences across subjects and time periods through parallel structures. Cai et al. (2024) proposed a multi-task, multi-branch spatio-temporal-spectral feature representation model based on CNNs, which effectively models the spatial, temporal, and spectral characteristics of MI-EEG signals. Zhao et al. (2019) transformed EEG signals into a three-dimensional electrode distribution representation and employed a multi-branch 3D CNN for feature extraction. Wang et al. (2023) developed a weighted multi-branch architecture to handle cross-subject data. Miao et al. (2025) proposed a model combining whitening and multi-scale feature fusion. In addition, filter bank multi-scale CNNs (Liu et al., 2023), attention-based multi-scale temporal convolutional networks (Huang et al., 2025), and frequency band attention-based temporal convolutional networks (Ma et al., 2025) have all incorporated multi-branch structures. Although these approaches capture MI-EEG features across different rhythms through multi-branch designs, they lack the ability to model global feature representations. Therefore, some studies have introduced temporal attention mechanisms to address this limitation.

Transformers were initially developed for sequence modeling in natural language processing and have recently been applied to MI-EEG decoding tasks to exploit temporal dependency information (Vaswani et al., 2017). Compared with recurrent neural networks (RNN) and long short-term memory (LSTM) networks (Hochreiter and Schmidhuber, 1997), Transformers are more suitable for high-dimensional, multi-channel time-series data such as MI-EEG signals. Altaheri et al. (2023) proposed an attention-based temporal convolutional network that significantly improves classification performance with fewer parameters. Song et al. (2023) developed EEG Conformer, which integrates local and global features within a unified framework. Luo et al. (2024) constructed a multi-branch fusion Transformer achieving an average accuracy of 86.93%. Liang et al. (2024) designed the cnnCosMSA model based on CNN and CoS attention, alleviating the attention collapse problem. Deng et al. (2025) proposed a network integrating CNN and Swin Transformer, which effectively handles high-dimensional and low signal-to-noise ratio EEG signals. Miao et al. (2026) employed a multi-head attention Transformer encoder for feature modeling, enhancing the capture of global dependencies.

In summary, the integration of CNNs with temporal attention mechanisms has become a mainstream trend. However, existing multi-branch CNN architectures typically enhance feature diversity through feature concatenation or element-wise addition, while neglecting the relative importance of features from different branches. This may introduce redundant information and makes it difficult to effectively represent the non-stationary and highly individual-dependent of MI-EEG signals. To address this issue, this paper proposes a weighted multi-scale attention-based temporal convolutional network (WMA-TCNet). The proposed model employs a Global-aware Scale Attention (GSA) module to adaptively recalibrate feature weights, thereby optimizing multi-branch feature fusion. In addition, a Temporal Attention Module (TAM) is introduced to guide the TCN in integrating local and global features. This design enhances MI-EEG decoding performance without significantly increasing model complexity. The main contributions of this study are as follows.

1. Global-aware Scale Attention: The overall temporal-spatial information of each branch is compressed to generate features with global awareness. A lightweight gating network is then applied to adaptively recalibrate the features from different branches, enabling dynamic enhancement of rhythm-specific information and suppression of redundant information across branches.
2. Channel-Preserving Prior Path (CP3): The original channel features are directly incorporated into the subsequent feature representation. This effectively alleviates the attenuation of electrode correlation caused by multi-scale convolutions and provides a stable input for spatial modeling.
3. Temporal Attention Module: This module employs two-dimensional convolution to model inter-sample relationships from both the global response trend and local salient activations of the time series. The learned attention weights are then used to emphasize important temporal segments.

## 2 Methods

### 2.1 Overview of WMA-TCNet

**FIGURE 1:**
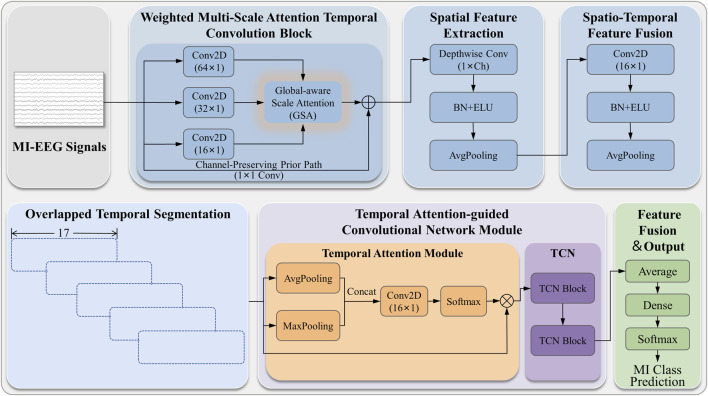
Architecture of the WMA-TCNet model.

The architecture of the WMA-TCNet model is illustrated in Figure 1. The design of this architecture is inspired by the temporal dependency, inter-subject variability, and electrode channel correlations of MI-EEG signals. Its primary objective is to capture the complex temporal characteristics of MI-EEG signals through effective feature selection and fusion. The overall framework consists of six key components: the Weighted Multi-scale Attention Temporal Convolution Block (WMATCB), Spatial Feature Extraction (SFE), Spatio-temporal Feature Fusion (STFF), Overlapped Temporal Segmentation (OTS), Temporal Attention-guided Convolutional Network Module (TACNM), and Feature Fusion and Output (FFO).

MI-EEG signals are first fed into the Weighted Multi-scale Attention Temporal Convolution Block. This module consists of parallel multi-scale temporal convolutions, GSA, and CP3, and is designed to fully exploit temporal features at different scales in EEG signals. Multiple convolution kernels with different receptive fields are employed to simultaneously capture short-term transient features and long-term rhythmic information from MI-EEG signals. Subsequently, GSA performs weighted fusion of features from different scale branches, enabling the network to dynamically adjust the attention to different temporal scales according to the temporal characteristics of the input samples, while suppressing redundant information. In addition, CP3 preserves the original channel information to a certain extent during the fusion process. This module generates low-level feature representations with enhanced expressive capability, providing a stable and effective foundation for subsequent spatial feature extraction and deep feature learning.

After obtaining the above features, the Spatial Feature Extraction module is applied to explicitly learn the spatial dependencies among electrode channels. This enables the model to preserve temporal features while capturing spatial distribution patterns related to motor imagery. Subsequently, the Spatio-temporal Feature Fusion module further integrates temporal and spatial information. This module performs convolution along the temporal dimension and expands channel representations, thereby generating more discriminative and compact spatio-temporal feature representations. For the spatio-temporal features, overlapped temporal segmentation is adopted to segment the feature sequence into n overlapping temporal segments, which are then fed in parallel into subsequent modules. This design allows the model to repeatedly learn dynamically changing EEG patterns at different temporal positions, thereby enhancing its ability to capture local information in non-stationary MI-EEG signals.

For intra-window features, TAM operates on two aspects—global response trends and local salient activations—by assigning weights to individual sample points, thereby guiding the network to focus on temporal segments relevant to motor imagery tasks. The TCN further extracts higher-level and more discriminative spatio-temporal dependencies from the weighted sequence features. Finally, the features are aggregated through average fusion and fed into a classifier, where a fully connected layer and Softmax function are used to perform probability prediction for MI classification. Table 1 presents the detailed parameter settings of each module in the WMA-TCNet model.

**TABLE 1:** Detailed parameter settings for each module of the WMA-TCNet model.

Block	Layer		Filters	Kernel size	Input	Output
WMATCB	Conv2D		F1	(64 × 1)	( T , Ch , 1)	( T , Ch , F1 )
	Conv2D		F1	(32 × 1)	( T , Ch , 1)	( T , Ch , F1 )
	Conv2D		F1	(16 × 1)	( T , Ch , 1)	( T , Ch , F1 )
	GSA		​	​	( N , T , Ch , 1)	( T , Ch , F1 )
	CP3		F1	(1 × 1)	( T , Ch , 1)	( T , Ch , F1 )
SFE	Depthwise Conv		F2	(1× Ch )	( T , Ch , F1 )	( T , 1 , F2 )
	BN		​	​	( T , 1 , F2 )	( T , 1 , F2 )
	ELU		​	​	( T , 1 , F2 )	( T , 1 , F2 )
	AvgPooling		​	​	( T , 1 , F2 )	( T/8 , 1 , F2 )
STFF	Conv2D		F2	(16 × 1)	( T/8 , 1 , F2 )	( T/8 , 1 , F2 )
	BN		​	​	( T/8 , 1 , F2 )	( T/8 , 1 , F2 )
	ELU		​	​	( T/8 , 1 , F2 )	( T/8 , 1 , F2 )
	AvgPooling		​	​	( T/8 , 1 , F2 )	( T/56 , 1 , F2 )
OTS	​		​	​	( T/56 , 1 , F2 )	( $T^{\prime }$ , 1 , F2 )
TACNM	TAM	AvgPooling	​	​	( $T^{\prime }$ , 1 , F2 )	( $T^{\prime }$ , 1 , 1 )
		MaxPooling	​	​	( $T^{\prime }$ , 1 , F2 )	( $T^{\prime }$ , 1 , 1 )
		Concat	​	​	​	( $T^{\prime }$ , 1 , 2 )
		Conv2D	1	(16 × 1)	( $T^{\prime }$ , 1 , 2 )	( $T^{\prime }$ , 1 , 1 )
		Softmax	​	​	( $T^{\prime }$ , 1 , 1 )	( $T^{\prime }$ , 1 , 1 )
		Output	​	​	​	( $T^{\prime }$ , 1 , F2 )
	TCN	Reshape	​	​	( $T^{\prime }$ , 1 , F2 )	( $T^{\prime }$ , F2 )
		TCN block	​	​	( $T^{\prime }$ , F2 )	( $T^{\prime }$ , 32 )
		TCN block	​	​	( $T^{\prime }$ , 32 )	( $T^{\prime }$ , 32 )
		Output	​	​	( $T^{\prime }$ , 32 )	(1, 32 )
FFO	Average		​	​	( n , 1, 32 )	(1, 32 )
	Dense		​	​	(1, 32 )	$N_{class}$
	Softmax		​	​	$N_{class}$	$N_{class}$

### 2.2 Weighted multi-scale attention temporal convolution block

The design of this module is inspired by the use of convolution kernels with different receptive fields to extract multi-scale temporal features, which can capture distinct rhythmic characteristics of the signal (Qiu et al., 2026). Therefore, to enhance the model’s ability to perceive rhythms associated with different MI tasks and to dynamically fuse multi-scale temporal information, a Weighted Multi-scale Attention Temporal Convolution Block is proposed. This module performs adaptive selection and fusion of features from different temporal receptive fields through parallel multi-scale temporal convolutions and GSA. At the same time, a Channel-Preserving Prior Path is introduced to stabilize spatial feature representation to a certain extent. Let the input feature map be $X\in R^{T\times Ch\times 1}$ , where T denotes the number of sampling points and Ch denotes the number of electrode channels. First, multiple parallel temporal convolution branches are constructed. Each branch uses convolution kernels of different sizes $k_{i}\in \left\{k_{1},k_{2},\ldots \ldots ,k_{N}\right\}$ , with kernel size $k_{i}\times 1$ , to capture temporal features under different receptive fields, as shown in Equation 1:

$$F_{i}=\text{BN}\left({Conv2D}_{k_{i}\times 1}\left(X\right)\right),i=1,\ldots \ldots ,N$$ (1)

where BN (Ioffe and Szegedv, 2015) denotes the batch normalization operation, N represents the number of branches, and the number of output channels for all branches is set to F1.

**FIGURE 2:**
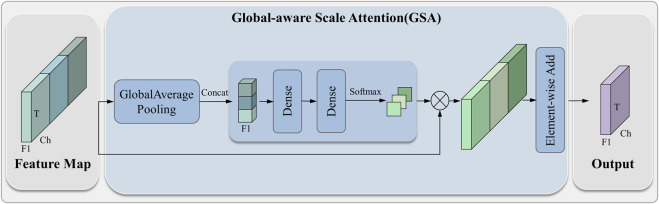
Compositional structure of GSA.

Subsequently, the multi-scale features are adaptively fused using the GSA mechanism. The structure of GSA is illustrated in Figure 2. First, global average pooling is applied to the output $F_{i}$ of each branch to obtain scale-level feature representations with global awareness, as shown in Equation 2:

$$z_{i}=\text{GAP}\left(F_{i}\right)$$ (2)

Then, as shown in Equation 3, the feature representations from all scales are concatenated and fed into a gated attention network. Through a two-layer fully connected structure, attention weights corresponding to each scale are generated:

$$\alpha =\text{Softmax}\left(\text{gate}\left(z_{1},\ldots \ldots ,z_{N}\right)\right)$$ (3)

where $\alpha =\left[\alpha_{1},\ldots \ldots ,\alpha_{N}\right]$ , and $\sum_{i}\alpha_{i}=1$ .

Based on the above attention weights, the original output feature maps of each scale branch are weighted and then summed element wise to obtain the fused multi-scale feature, as shown in Equation 4:

$$F_{\text{ms}}=\sum_{i=1}^{N}\alpha_{i}\cdot F_{i}$$ (4)

To balance the information flow between original features and transformed features, and to mitigate the attenuation of electrode correlations caused by multi-scale convolutions, the module introduces a CP3 (Shafiq and Gu, 2022). This path applies a 1 × 1 convolution to map the channels of the MI-EEG signal and multiplies the result by a fixed coefficient λ (the value of λ is specified in Section 3.4.3), yielding the prior feature, as shown in Equation 5:

$$F_{c}=\lambda \cdot \text{BN}\left({Conv2D}_{1\times 1}\left(X\right)\right)$$ (5)

Finally, the fused multi-scale feature $F_{\text{ms}}$ and the prior feature $F_{c}$ are combined through element-wise addition, and the module output is obtained by applying the Exponential Linear Unit (ELU) (Clevert et al., 2016) activation function, as shown in Equation 6:

$$Y_{1}=\text{ELU}\left(F_{\text{ms}}+F_{c}\right)$$ (6)

Through this design, the Weighted Multi-scale Attention Temporal Convolution Block can dynamically adjust the attention to more discriminative scales with relatively few parameters, without increasing model complexity. This effectively enhances the model’s ability to represent complex temporal patterns in MI-EEG signals.

### 2.3 Spatial feature extraction

After obtaining the multi-scale temporal representations, a Spatial Feature Extraction module is introduced following the Weighted Multi-scale Attention Temporal Convolution Block to further explore the spatial topological relationships among multiple electrode channels in MI-EEG signals. This module is implemented based on depthwise separable convolution, which effectively captures spatial correlations between electrode channels while significantly reducing the number of parameters. The input feature map of this module is $Y_{1}\in R^{T\times Ch\times F1}$ . As shown in Equation 7, the Spatial Feature Extraction module employs depthwise separable convolution with a kernel size of 1×Ch , performing convolution independently along the spatial dimension for each feature channel. In this way, it explicitly models the spatial distribution characteristics across different EEG channels:

$$F_{Ch}={DepthwiseConv}_{1\times Ch}\left(Y_{1}\right)$$ (7)

The depth multiplier is set to D , expanding the feature dimension to F2=F1×D , thereby enhancing the model’s ability to represent complex spatial patterns. Subsequently, BN and the ELU activation function are sequentially applied to the spatial convolution output to stabilize the training process and improve the nonlinear representation capability of feature distributions. Finally, average pooling is performed along the temporal dimension to downsample local information, resulting in a more compact spatial feature representation. The final output of SFE is then given by Equation 8:

$$Y_{2}={\text{AvgPooling}2D}_{8\times 1}\left(\text{ELU}\left(\text{BN}\left(F_{Ch}\right)\right)\right)$$ (8)

### 2.4 Spatio-temporal feature fusion

To achieve deep integration of temporal and spatial feature information, a Spatio-temporal Feature Fusion module is introduced after the Spatial Feature Extraction module. This module performs modeling across the temporal dimension on the extracted spatial features, thereby obtaining fused spatio-temporal representations. The input feature map of this module is $Y_{2}\in R^{T/8\times 1\times F2}$ . As shown in Equation 9, a two-dimensional convolution with a kernel size of 16 × 1 is applied to extract local spatio-temporal fusion features:

$$F_{TC}={Conv2D}_{16\times 1}\left(Y_{2}\right)$$ (9)

By performing linear combinations along the feature channel dimension, features from different spatial filters are integrated to form spatio-temporal representations with higher-level semantic information. According to Equation 10, BN, ELU activation function, and average pooling are sequentially applied to the features, resulting in the final spatio-temporal fusion features:

$$Y_{3}={\text{AvgPooling}2D}_{7\times 1}\left(\text{ELU}\left(\text{BN}\left(F_{TC}\right)\right)\right)$$ (10)

### 2.5 Overlapped temporal segmentation

After spatio-temporal feature fusion, an overlapping temporal segmentation strategy is introduced to improve the model’s robustness to non-stationary EEG signals. This mechanism segments the feature sequence along the temporal dimension with overlap. The input feature map is $Y_{3}\in R^{T/56\times 1\times F2}$ . A sliding window strategy with a stride of 1 is applied along the temporal dimension to divide the feature sequence into n overlapping subsequences, as shown in Equation 11, where each subsequence can be expressed as:

$$T_{i}=Y_{3}\left[i:i+T^{\prime }\right],i=1,2,\ldots \ldots ,n$$ (11)

where $T^{\prime }=T/56-n+1$ denotes the length of each individual window.

There is significant overlap between adjacent windows, ensuring smooth transitions of information across consecutive temporal segments and facilitating the model’s ability to capture local temporal dependencies. Each windowed feature subsequence is independently fed into the subsequent TACNM for further processing.

### 2.6 Temporal attention-guided convolutional network module

#### 2.6.1 Temporal attention module

**FIGURE 3:**
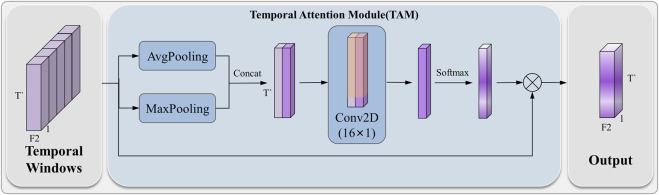
Compositional structure of TAM.

To emphasize discriminative temporal segments within each window and suppress redundant or noisy temporal information, TAM is introduced before the TCN, as illustrated in Figure 3. This module adaptively weights temporal features to guide the TCN to focus on important time steps. The input feature map of this module is $T_{i}\in R^{T^{\prime }\times 1\times F2}$ . First, according to Equations 12, 13, average pooling and max pooling are applied along the channel dimension to capture the global response trend and local salient activations, respectively:

$$X_{avg}^{i}=\text{Mean}\left(T_{i},axis=F2\right),i=1,2,\ldots \ldots ,n$$ (12)

$$X_{\max}^{i}=\mathrm{Max}\left(T_{i},axis=F2\right),i=1,2,\ldots \ldots ,n$$ (13)

Subsequently, the two types of features are concatenated along the channel dimension to form a joint descriptive representation. A two-dimensional convolution with a kernel size of $k_{t}\times 1$ is then applied to capture temporal dependencies and generate temporal attention. To ensure comparability of weights across different time positions, the resulting attention weights are normalized along the temporal dimension using the Softmax function, as shown in Equation 14:

$$\beta_{i}=\text{Softmax}\left({Conv2D}_{k_{t}\times 1}\left(C\text{oncat}\left({X_{avg}^{i};X}_{\max}^{i}\right)\right)\right),i=1,2,\ldots \ldots ,n$$ (14)

As shown in Equation 15, finally, the normalized temporal attention weights are multiplied element-wise with the original input features to achieve weighted modulation of the time series.

$$Y_{att}^{i}=T_{i}⊙\beta_{i},i=1,2,\ldots \ldots ,n$$ (15)

#### 2.6.2 Temporal convolutional network

**FIGURE 4:**
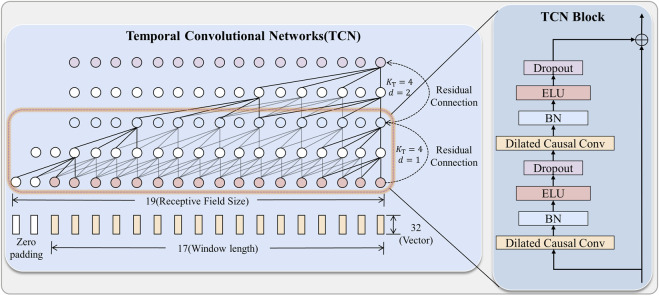
Compositional structure of TCN.

Guided by the TAM, a Temporal Convolutional Network is employed to perform deep temporal feature extraction on the weighted time features, aiming to capture both short-term and long-term temporal dependencies in MI-EEG signals. The input feature of this module is $Y_{att}^{i}\in R^{T^{\prime }\times 1\times F2}$ . The TCN consists of two stacked TCN Blocks, and each TCN Block is composed of two dilated causal convolution layers. Each convolution layer is followed by batch normalization and the ELU activation function (Lea et al., 2017). The structure of this module is illustrated in Figure 4.

In each TCN Block, causal convolution with a kernel size of $K_{T}=4$ is employed. An exponentially increasing dilation factor d is used to progressively expand the receptive field size (RFS):

$$RFS=1+2\left(K_{T}-1\right)\left(2^{m}-1\right)$$ (16)

where m denotes the total number of TCN Blocks.

Each dilated convolution layer in the TCN module is configured with 32 filters, and the dilation factors d of the two convolutional layers are set to 1 and 2, respectively. According to Equation 16, the temporal receptive field size of the TCN is calculated to be 19, indicating that the network can effectively model temporal dependencies spanning up to 19 consecutive time steps. Under the aforementioned network structure, the input sequence length to the TCN is 17, with each time step corresponding to a 32-dimensional feature vector. Since the input sequence length is smaller than the receptive field size of the TCN, the entire temporal sequence can be fully covered, thereby avoiding the loss of temporal information due to insufficient receptive field.

The TCN module outputs the feature representation corresponding to the final time step of the sequence, with a dimension of 32. For the n subsequences obtained from the sliding time window segmentation, the output features extracted independently by the TCN for each window are averaged for fusion. The fused feature vector is then fed into a fully connected layer, which contains $N_{class}$ neurons corresponding to all categories in the MI task. Finally, the Softmax function is used to output the predicted probabilities for each class.

## 3 Experimental design

### 3.1 Datasets

In this study, to comprehensively evaluate the performance of the proposed model, two publicly available BCI competition datasets were selected, namely the BCI Competition IV-2a dataset and the BCI Competition IV-2b dataset. These datasets cover various MI tasks and experimental paradigms. The detailed information of the datasets is presented in Table 2.

**TABLE 2:** Dataset details.

Datasets	Subjects	MI task	Ch	Time points	MI trails
BCI-2a	9	Left hand, Right hand, foot,Tongue	22	1125	5184
BCI-2b	9	Left hand, Right hand	3	1125	6480

#### 3.1.1 BCI-2a

**FIGURE 5:**
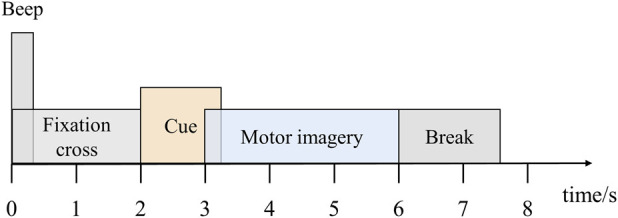
Acquisition process of the BCI Competition IV-2a dataset.

The BCI Competition IV-2a dataset (Tangermann et al., 2012), released by Graz University of Technology in 2008, is a widely used benchmark dataset in the field of MI-EEG decoding. This dataset contains MI-EEG data from nine healthy subjects. Each subject completed two separate experimental sessions on different days, resulting in a total of 576 trials. In the experimental setup, one session is randomly selected for model training, while the other session is used for model testing. EEG signals were recorded using 25 Ag/AgCl electrodes, among which three electrooculogram channels were excluded from subsequent analysis. Each MI trial lasts for 4 s, with a sampling rate of 250 Hz. The signals were band-pass filtered within the range of 0.5–100 Hz after acquisition. The experimental tasks include four MI classes: left hand, right hand, both feet, and tongue. The trial acquisition procedure is illustrated in Figure 5.

#### 3.1.2 BCI-2b

The BCI Competition IV-2b dataset (Tangermann et al., 2012), released during the fourth BCI Competition, is primarily designed for binary MI classification tasks. This dataset contains left-hand and right-hand MI-EEG data from nine subjects. Each subject participated in five experimental sessions, among which the first three sessions are used as the training set, comprising 400 trials, and the remaining two sessions are used as the testing set, comprising 320 trials. EEG signals were recorded using three channels. Each MI trial lasts for 4 s, with a sampling rate of 250 Hz. The signals were processed with a 0.5–100 Hz band-pass filter followed by a 50 Hz notch filter to suppress power-line noise interference.

### 3.2 Experimental details

The experiments were conducted using the TensorFlow 2.18.0 framework with Python version 3.12. The experimental environment was based on the Windows 11 operating system. The computational hardware included an Intel Core i5-14600KF CPU and an NVIDIA GeForce GTX 4060 GPU. The batch size was set to 64, and the learning rate was set to 0.001. The Adam optimizer was employed to optimize model parameters, and the loss function was defined as cross-entropy. To reduce redundant training and accelerate convergence, the maximum number of training epochs was set to 1000. In addition, L2 regularization and an early stopping mechanism were applied.

### 3.3 Evaluation metrics

Acc and the Kappa coefficient are adopted as performance evaluation metrics for the proposed network. Acc reflects the proportion of correctly classified samples, while the Kappa coefficient measures the reliability of the classifier’s predictions. These metrics are commonly used in EEG decoding tasks and have been theoretically and empirically validated as effective measures for evaluating classification performance (Schirrmeister et al., 2017). The corresponding formulations are given in Equations 17 and 18, respectively.

$$Acc=\frac{TP+TN}{TP+FN+FP+TN}$$ (17)

In Equation 17, TP denotes true positives, representing the number of trials correctly identified as the target class. FP denotes false positives, representing the number of non-target trials incorrectly classified as the target class. TN denotes true negatives, representing the number of trials correctly identified as non-target classes. FN denotes false negatives, representing the number of target trials that were not correctly identified.

$$Kappa=\frac{p_{o}-p_{e}}{1-p_{e}}$$ (18)

In Equation 18, $p_{o}$ represents the overall classification accuracy, defined as the ratio of the total number of correctly classified samples to the total number of samples. $p_{e}$ denotes the expected agreement, calculated as the sum of the products of the true and predicted sample counts for each class divided by the square of the total number of samples.

$$p_{o}=\frac{\sum_{i=1}^{N_{class}}S_{i}}{M}$$ (19)

$$p_{e}=\frac{\sum_{i=1}^{N_{class}}a_{i}*b_{i}}{M^{2}}$$ (20)

In Equation 19 and Equation 20, $N_{class}$ denotes the total number of classes, M represents the total number of samples across all classes, and $S_{i}$ denotes the number of samples in class i that are correctly classified. $a_{i}$ and $b_{i}$ represent the numbers of true samples and predicted samples for class i , respectively.

### 3.4 Experimental results and analysis

To validate the effectiveness of the proposed model, a series of ablation studies and comparative experiments were conducted. In addition, further visualization analyses were performed on the network. In the following experiments, raw MI-EEG signals were directly fed into the model without additional preprocessing, in order to preserve the authenticity of the data.

#### 3.4.1 Ablation study

This section presents ablation experiments conducted on the BCI-2a and BCI-2b datasets. By individually removing key components of the model, including GSA, CP3, OTS, TAM, and TCN, the contribution of each module to the overall performance is analyzed.

As shown in Table 3, the complete WMA-TCNet achieves the best performance on the BCI-2a dataset. When each core component is removed, the classification performance consistently decreases. Specifically, removing GSA results in a 3.3% drop in accuracy; removing the CP3 leads to a 0.9% decrease; removing OTS causes a 4.7% reduction; removing TAM results in a 2.3% decrease; and removing TCN leads to the largest drop of 6.2% in accuracy. The complete WMA-TCNet also achieves the best performance on the BCI-2b dataset. After removing GSA, the accuracy decreases by 0.4%; removing the CP3 leads to a 0.8% reduction; removing OTS results in a 0.5% decrease; removing TAM causes a 0.4% drop; and removing TCN leads to a 3.8% decrease in accuracy. The ablation results on both datasets demonstrate the rationality and effectiveness of each component in the WMA-TCNet model. The modules exhibit strong complementarity, and their joint design enables WMA-TCNet to achieve superior performance across different subjects and datasets.

**TABLE 3:** Ablation study of the WMA-TCNet model on the BCI-2a dataset and BCI-2b dataset.

Block	BCI-2a		BCI-2b	
	Avg(%)	Kappa	Avg(%)	Kappa
WMA-TCNet	**85.8**	**0.807**	**90.0**	**0.796**
Without GSA	82.5	0.764	89.6	0.791
Without CP3	84.9	0.796	89.2	0.783
Without OTS	81.1	0.747	89.5	0.790
Without TAM	83.5	0.778	89.6	0.793
Without TCN	79.6	0.729	86.2	0.714

Bold highlights the best decoding accuracies.

**FIGURE 6:**
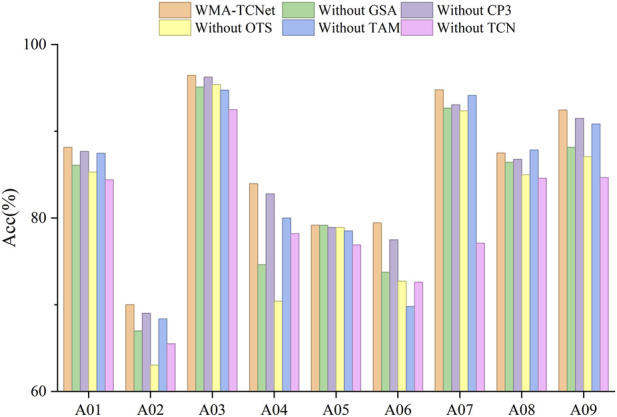
Comparison chart of ablation study of the WMA-TCNet model on the BCI-2a dataset.

**FIGURE 7:**
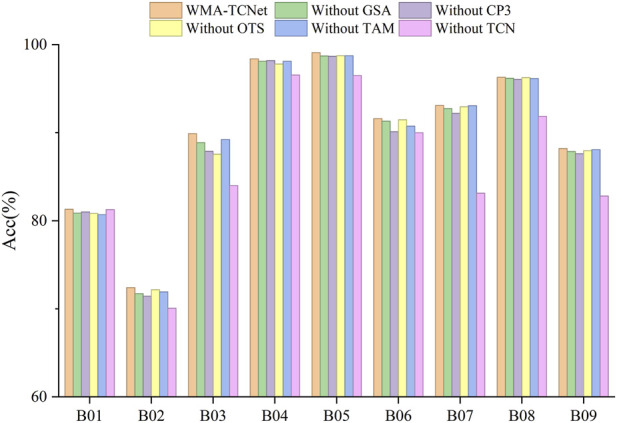
Comparison chart of ablation study of the WMA-TCNet model on the BCI-2b dataset.

Figures 6, 7 present in detail the impact of each core module on the classification accuracy of individual subjects in the two datasets. The results indicate that removing any core component from the model leads to a decline in classification performance for almost all subjects. The WMA-TCNet model achieves the best performance for all subjects except A05 in the BCI-2a dataset and B08 in the BCI-2b dataset.

Meanwhile, the ablation experiments show that removing the TCN has the greatest impact on model performance. This phenomenon is mainly determined by the unique role of the TCN in the overall architecture. Specifically, the TCN is responsible for integrating the feature sequences extracted by the preceding modules. Therefore, the model performance can be fully realized only when sufficient temporal modeling is conducted by the TCN.

#### 3.4.2 Comparison of attention mechanisms

To evaluate the effectiveness of different attention mechanisms in guiding the network to focus on MI-related temporal segments, this section compares the performance of models without attention, with TAM, and with MSA. For MSA, the number of heads is set to 2, and the head dimension is set to 8. As shown in Table 4, the introduction of attention mechanisms improves model performance in all cases. Among them, TAM achieves the best performance, with an increase of 2.3% in accuracy and 0.029 in Kappa. In comparison, although MSA also improves performance, it significantly increases the number of parameters. These results indicate that TAM can enhance model performance with fewer additional parameters, making it more suitable for MI-EEG temporal modeling tasks.

**TABLE 4:** Impact of different attention mechanisms on the performance of the WMA-TCNet model.

Attention mechanism	Parameters	Avg(%)	Kappa
Without attention	105.3k	83.5	0.778
With TAM	105.5k	**85.8**	**0.807**
With MSA	116.3k	84.6	0.795

Bold highlights the best decoding accuracies.

#### 3.4.3 Parameter sensitivity analysis

**FIGURE 8:**
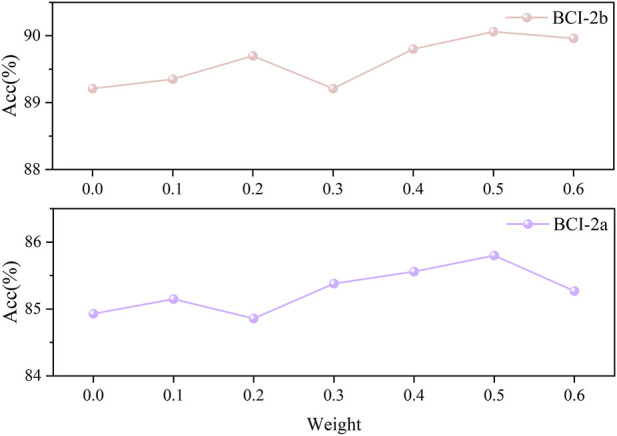
Impact of weights λ on model performance across two datasets.

To evaluate the impact of the weight parameter λ in CP3 on model performance, systematic experiments were conducted on both datasets. The variation of classification accuracy with respect to λ is illustrated in Figure 8.

CP3 directly incorporates the original channel features into the fused feature representation, with the contribution controlled by the parameter λ . This design allows the model to preserve physiologically meaningful electrode channel distribution information from the original MI-EEG signals while extracting multi-scale temporal features, thereby providing more reliable input for subsequent spatial feature extraction.

The experimental results indicate that when the parameter λ is set to 0, the model performance remains at a relatively low level. As λ gradually increases, the classification performance on both datasets exhibits a trend of first increasing and then decreasing. When λ is within a small range, its contribution to feature fusion is limited, and the model mainly relies on multi-scale temporal features. As λ increases, the proportion of original features in the fused representation also increases, which helps enhance the stability of spatial structural information. Consequently, the model achieves relatively better classification performance on both the BCI-2a and BCI-2b datasets.

However, when the parameter λ increases further, the model performance begins to decline. This phenomenon indicates that an excessively large λ causes the original features to dominate the fused representation, thereby weakening the discriminative capability of multi-scale temporal features. In this case, some discriminative features are overshadowed by shallow original features, limiting the model’s ability to capture complex temporal dynamics and ultimately degrading the overall classification performance.

Overall, the analysis indicates that CP3 helps achieve a balance between multi-scale temporal features and the preservation of original channel information. However, an excessively large λ may suppress the contribution of multi-scale features. The results show that when λ is set within the range of 0.4–0.5, the model achieves relatively optimal performance on both datasets. Therefore, λ = 0.5 is adopted in this study.

#### 3.4.4 Subject-dependent experiments

In this section, the performance of the WMA-TCNet model is evaluated on the BCI-2a and BCI-2b datasets under a subject-dependent setting. The proposed method is compared with several existing models, including ShallowConvNet (Schirrmeister et al., 2017), DeepConvNet (Schirrmeister et al., 2017), EEGNet (Lawhern et al., 2018), MBEEG_SENet (Altuwaijri et al., 2022), ATCNet (Altaheri et al., 2023), EA-EEG (Miao et al., 2025), and TFCA-TransNet (Miao et al., 2026). The results are presented in Table 5.

**TABLE 5:** Subject-dependent performance comparison results of the model on the BCI-2a dataset.

Methods	A01	A02	A03	A04	A05	A06	A07	A08	A09	Avg(%)	Kappa
ShallowConvNet	84.4	**75.5**	92.5	58.2	80.9	72.6	77.1	**90.6**	83.7	79.5*	0.727
DeepConvNet	77.1	74.1	77.1	71.9	81.8	77.8	76.9	79.0	79.9	77.3*	0.697
EEGNet	80.9	54.5	88.9	61.8	71.5	54.5	86.8	83.7	80.6	73.7**	0.649
MBEEG_SENet	89.4	63.0	94.8	70.0	77.1	68.6	63.4	89.8	85.4	77.9*	0.706
ATCNet	90.1	70.8	**96.9**	81.2	79.9	73.6	93.5	87.5	92.4	85.1	0.805
EA-EEG	**90.7**	65.0	96.7	81.2	**83.0**	60.9	94.2	85.3	83.7	82.3	0.762
TFCA-TransNet	88.2	64.4	**96.9**	82.6	77.1	71.5	94.5	90.1	86.8	83.6*	0.779
Proposed	88.2	70.0	96.4	**84.0**	79.2	**79.5**	**94.8**	87.5	**92.5**	**85.8**	**0.807**

Bold highlights the best decoding accuracies. The * and ** indicate that the decoding accuracies of WMA-TCNet, are significantly higher than those of the compared methods with p < 0.05 and p < 0.01, respectively.

From the overall performance perspective, methods based solely on convolutional structures exhibit certain limitations in both average classification accuracy and Kappa coefficient. In contrast, models incorporating attention mechanisms achieve superior performance on most subjects, indicating that attention mechanisms play a positive role in highlighting discriminative temporal or spatial features. Compared with the aforementioned methods, WMA-TCNet achieves an average classification accuracy of 85.8% on the BCI-2a dataset, outperforming all compared approaches in overall performance. In particular, compared with ATCNet and TFCA-TransNet, the proposed method demonstrates better classification results across multiple subjects.

**FIGURE 9:**
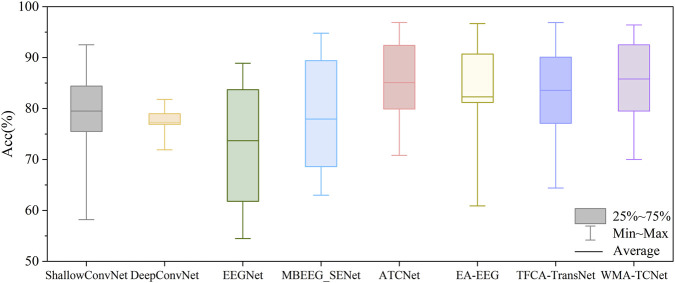
Comparison boxplot of the model on the BCI-2a dataset.

In addition, box plots are employed to perform a statistical analysis of classification accuracy across different methods, including the proposed approach. Box plots provide an intuitive visualization of the average performance, variability, and distribution of extreme values. Figure 9 presents the box plot comparison of different methods on the BCI-2a dataset, where each method is represented by a box of a different color. The horizontal line inside each box indicates the mean value, the whiskers represent the minimum and maximum values, and the height of the box corresponds to the interquartile range, reflecting the middle 50% of the data. Compared with other methods, the proposed WMA-TCNet exhibits a higher average accuracy and a more compact interquartile range in the box plot. Moreover, the whiskers are relatively more convergent, indicating fewer subjects with extremely low performance.

As shown in Table 6, WMA-TCNet achieves an average classification accuracy of 90.0% on the BCI-2b dataset, outperforming most of the compared models in overall performance and obtaining the best or second-best results across multiple subjects. Although TFCA-TransNet achieves higher accuracy on certain individual subjects, its overall performance exhibits greater variability. In contrast, WMA-TCNet maintains a high average accuracy while achieving a more balanced performance distribution across subjects.

**TABLE 6:** Subject-dependent performance comparison results of the model on the BCI-2b dataset.

Methods	B01	B02	B03	B04	B05	B06	B07	B08	B09	Avg(%)	Kappa
ShallowConvNet	72.5	65.4	79.4	97.5	92.8	90.0	86.9	92.8	85.9	84.8**	0.696
DeepConvNet	71.6	**73.6**	87.2	96.6	93.4	84.1	87.8	92.2	81.9	85.4**	0.707
EEGNet	77.2	69.3	88.4	98.8	98.1	90.6	93.1	94.7	91.3	89.1	0.781
MBEEG_SENet	**81.3**	70.0	86.9	98.4	97.8	90.3	91.9	95.3	**92.2**	89.3	0.787
ATCNet	80.8	71.9	88.8	98.4	97.8	90.5	94.7	94.7	87.0	89.4	0.793
EA-EEG	72.8	64.9	87.8	98.5	98.9	86.1	**94.9**	92.2	86.8	87.0*	0.725
TFCA-TransNet	**81.3**	72.7	87.3	**99.4**	**99.1**	87.9	93.2	95.6	81.7	88.7	0.755
Proposed	**81.3**	72.4	**89.9**	98.4	**99.1**	**91.6**	93.1	**96.3**	88.2	**90.0**	**0.796**

Bold highlights the best decoding accuracies. The * and ** indicate that the decoding accuracies of WMA-TCNet, are significantly higher than those of the compared methods with p < 0.05 and p < 0.01, respectively.

**FIGURE 10:**
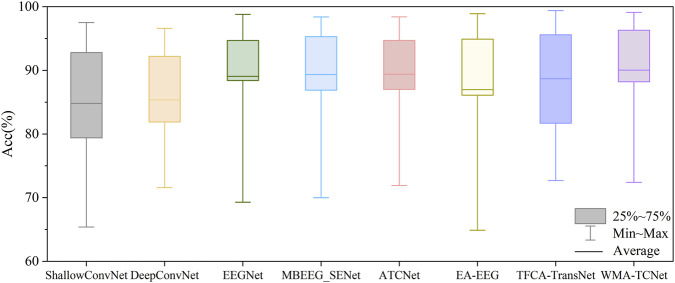
Comparison boxplot of the model on the BCI-2b dataset.

In addition, box plots are used to statistically analyze the classification accuracy of different methods, including the proposed approach, on the BCI-2b dataset, as shown in Figure 10. Compared with other methods, WMA-TCNet maintains more stable and higher overall classification performance in the binary classification task.

To further evaluate the trade-off between model complexity and performance of the proposed WMA-TCNet, Table 7 presents a comparison of the number of model parameters across different methods. The results demonstrate that WMA-TCNet achieves a more balanced compromise between parameter scale and classification performance. Specifically, WMA-TCNet has 105.5k parameters, which is significantly lower than that of DeepConvNet and TFCA-TransNet. Compared with ATCNet, it not only reduces the number of parameters but also achieves superior classification performance. These results indicate that WMA-TCNet does not rely on simply increasing network depth or introducing a large number of parameters to improve performance, but instead achieves effective discriminative feature extraction through a more efficient architectural design.

**TABLE 7:** Comparative analysis with existing methods.

Methods	Parameters	Inference time	FLOPs	Avg(%)	Kappa
ShallowConvNet	46.1k	37.98 ± 0.93 ms	127.3M	79.5	0.727
DeepConvNet	283.3k	38.85 ± 1.56 ms	227.1M	77.3	0.697
EEGNet	**2.6k**	**37.14 ± 1.04 ms**	**26.7M**	73.7	0.649
MBEEG_SENet	10.5k	38.26 ± 1.03 ms	71.6M	77.9	0.706
ATCNet	115.2k	40.85 ± 1.99 ms	82.3M	85.1	0.805
EA-EEG	35.3k	39.81 ± 1.07 ms	55.1M	82.3	0.762
TFCA-TransNet	253.3k	41.38 ± 1.99 ms	397.8M	83.6	0.779
Proposed	105.5k	40.13 ± 1.16 ms	102.7M	**85.8**	**0.807**

Bold highlights the optimal values of all indicators.

In addition, inference latency is also a key factor that affects the performance of real-time MI devices. On the same standard hardware platform, the single-sample inference latency of WMA-TCNet is 40.13 ± 1.16 ms. Although this latency is comparable to that of several existing mainstream models, there is still room for further improvement compared with the lightweight EEGNet model. In terms of computational cost, WMA-TCNet requires 102.7M FLOPs, imposing a relatively moderate computational burden.

#### 3.4.5 Cross-subject experiments

To evaluate the generalization capability of WMA-TCNet in cross-subject scenarios, cross-subject experiments were further conducted on the BCI-2a and BCI-2b datasets. A leave-one-subject-out strategy was adopted, where the data from one subject was used as the test set, and the data from all remaining subjects were used for model training. This process was repeated until all subjects had been evaluated once. As shown in Table 8, in the four-class classification task on the BCI-2a dataset, WMA-TCNet achieves an average accuracy of 68.6%, with a corresponding Kappa coefficient of 0.581, demonstrating strong generalization capability and robustness. In the binary classification task on the BCI‐2b dataset, WMA‐TCNet, also outperforms all compared models, achieving an average accuracy of 79.5% and a Kappa coefficient of 0.590. Compared with the second-best model, MBEEG_SENet, it achieves an improvement of 3.4%.

**TABLE 8:** Cross-subject performance comparison results of the model on the BCI-2a dataset and BCI-2b dataset.

Methods	BCI-2a		BCI-2b	
	Avg(%)	Kappa	Avg(%)	Kappa
ShallowConvNet	65.1	0.536	73.5	0.467
DeepConvNet	62.0	0.498	73.9	0.474
EEGNet	64.8	0.528	75.2	0.503
MBEEG_SENet	60.2	0.467	76.1	0.520
TFCA-TransNet	66.3	0.552	75.8	0.516
Proposed	**68.6**	0.581	**79.5**	0.590

Bold highlights the best decoding accuracies.

#### 3.4.6 Visualization

**FIGURE 11:**
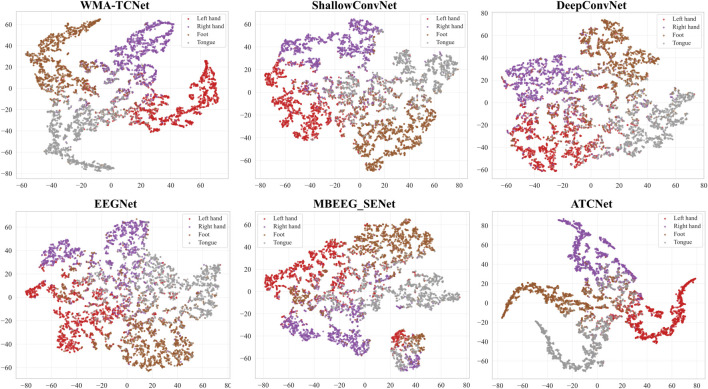
t-SNE-based feature visualization analysis.

To intuitively compare the ability of different methods in capturing discriminative features, the t-SNE algorithm is employed to embed the high-dimensional features learned by each model into a low-dimensional space for visualization. The results are shown in Figure 11. It can be observed that the features extracted by WMA-TCNet exhibit more compact intra-class clustering, along with clearer class boundaries and higher inter-class separability. In contrast, the features extracted by ShallowConvNet, DeepConvNet, EEGNet, and MBEEG_SENet are more scattered, showing weaker intra-class compactness and inter-class separability. In particular, MBEEG_SENet and EEGNet present noticeable feature overlap between different classes, which limits their discriminative capability. Although ATCNet forms relatively compact intra-class clusters, its inter-class separability remains insufficient, with less distinct class boundaries. These results indicate that WMA-TCNet is more effective in capturing discriminative features of MI-EEG signals, leading to superior classification performance.

**FIGURE 12:**
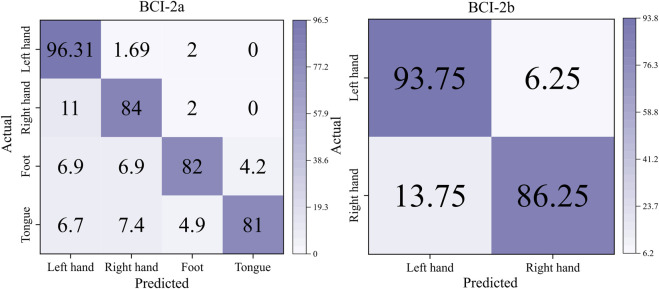
Confusion matrices for the two datasets.

As shown in Figure 12, the average confusion matrices of prediction results across all subjects for both datasets are presented to further analyze the classification performance of the model on different MI categories.

For the BCI-2a dataset, the classification accuracy for left-hand motor imagery is the highest, reaching 96.31%. In contrast, the accuracies for both feet and tongue motor imagery are relatively lower, and these classes are often misclassified as left-hand or right-hand movements. This indicates a certain degree of similarity between these classes and hand movements in the feature space, leading to increased classification difficulty. For the BCI-2b dataset, the classification accuracy for left-hand motor imagery is also the highest, reaching 93.75%, while the accuracy for right-hand motor imagery is 86.25%, with frequent misclassification as left-hand movements. This confusion between left-hand and right-hand motor imagery is common in MI-EEG classification tasks and may be attributed to the similarity in activation patterns between the corresponding brain regions.

**FIGURE 13:**
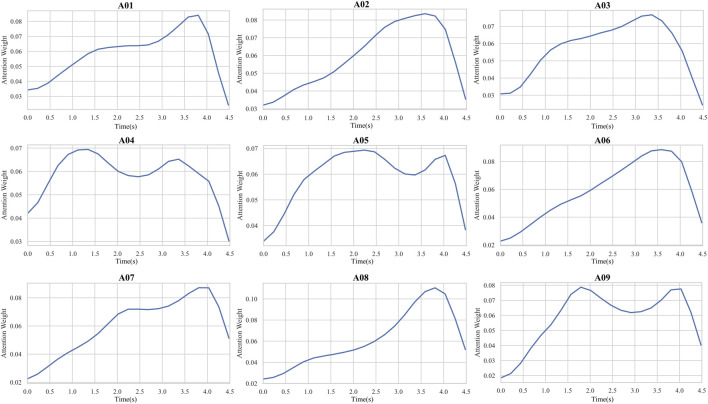
Distribution of temporal attention weights for the 9 subjects in the BCI-2a dataset.

Figure 13 illustrates the distribution of temporal attention weights for nine subjects over a 4.5 s MI trial (corresponding to 1.5 s–6 s as shown in Figure 5). According to the experimental paradigm, 0–0.5s corresponds to the fixation cross stage, 0.5–1.5s to the cue stage, and 1.5–4.5s to the MI stage. Overall, the temporal attention weights of all subjects exhibit clear stage-wise differences.

During the fixation cross stage, the attention weights remain at a generally low level with relatively smooth variations, indicating that the model pays limited attention to MI-EEG signals at this stage. This is consistent with the experimental design, where this stage is mainly used to stabilize the subject’s state and lacks explicit task-related neural activity.

After entering the cue stage, the attention weights of most subjects gradually increase. This increasing trend is relatively smooth for most subjects, while for a few subjects (e.g., A04 and A05), a more abrupt rise is observed, reflecting inter-subject variability in neural responses during the cue stage. During the MI stage, all nine subjects exhibit significantly higher attention weights compared to the previous two stages, with peak attention values mainly occurring in the middle to late period of the MI stage. This phenomenon is highly consistent across all subjects, indicating that the model primarily relies on discriminative features generated during the MI stage rather than early non-task-related or preparatory signals. Moreover, the attention weights decrease rapidly toward the end of the MI stage for all subjects, suggesting a reduction in the intensity of motor imagery as the trial approaches completion.

Although there are some differences among subjects in terms of peak attention values and their temporal locations, the overall pattern—enhanced attention during the MI stage and significantly reduced attention during non-MI stages—remains consistent across all subjects. These results demonstrate that the proposed temporal attention mechanism can effectively guide the TCN to focus on time intervals with clear neurophysiological relevance, even in the presence of inter-subject variability.

**FIGURE 14:**
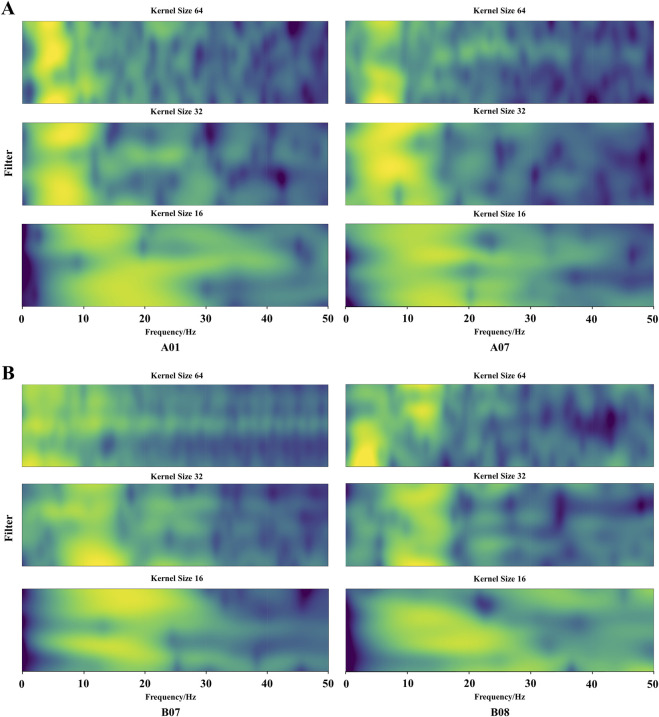
Filter responses of selected subjects from BCI-2a and BCI-2b. **(A)** A01 and A07. **(B)** B07 and B08.

As shown in Figure 14, the convolution kernels with sizes of 64 and 32 show stronger responses in the 4–14 Hz frequency band. This indicates that they are more effective in capturing low-frequency information related to the μ rhythm in MI-EEG signals. In contrast, the convolution kernel with a size of 16 exhibits a stronger response in the 10–30 Hz frequency band, which reflects its higher selectivity for the β rhythm and local high-frequency information. This pattern is consistent with the neurophysiological mechanism of MI tasks, namely the frequency bands associated with ERD/ERS phenomena. These results suggest that convolution kernels with receptive fields at different scales can provide complementary feature information in the frequency domain.

## 4 Discussion

The core innovations of WMA-TCNet are reflected in three key modules. First, the GSA module compresses and models the global temporal-spatial information across multiple branches, and employs a lightweight gating network to adaptively recalibrate the feature weights of each branch, thereby dynamically emphasizing scale-level information while suppressing redundancy. Second, the channel-preserving prior path retains the original channel structure during multi-scale feature fusion, effectively alleviating the attenuation of electrode correlations caused by multi-scale convolutions and providing a stable foundation for spatial feature modeling. Third, the TAM captures both the global response trends and local salient activations of the time series through two-dimensional convolution, guiding the TCN to accurately integrate local and global features.

The GSA module is designed to alleviate the limitations caused by equal-weight feature fusion in multi-scale temporal convolution. It is essentially a dynamic selection mechanism for multi-scale temporal features. It can select more discriminative scales according to the global response characteristics of the signal. Unlike the existing squeeze-and-excitation attention mechanism, GSA applies attention across different scales. It therefore emphasizes cross-scale contribution allocation rather than channel recalibration. In WMA-TCNet, each branch contains core neural oscillatory components related to MI. However, the discriminative capability of each rhythm may vary because of signal quality and inter-subject differences. GSA establishes a soft competition mechanism among the three effective branches. It assigns greater importance to the rhythmic branch with the clearest and most discriminative ERD/ERS patterns. At the same time, it relatively suppresses the contributions of branches that are more affected by noise or have weaker discriminative power, rather than removing them completely. This strategy ensures that the fused features are mainly guided by rhythmic information with higher signal quality. It also preserves the ability to extract complementary information from other rhythms. Compared with equal-weight fusion, this design improves the noise robustness and subject adaptability of the model.

The introduction of CP3 aims to compensate for the attenuation of inter-electrode channel correlation information caused by multi-scale temporal convolution. In MI-EEG signals, the relationships among electrode channels also contain discriminative information. However, when parallel multi-branch temporal convolution independently extracts temporal features at different scales, it inevitably weakens the original spatial structural correlations among channels. As a result, the subsequent SFE module may model distorted channel relationships, which can reduce spatial discriminability. Therefore, CP3 injects prior features into the fused features and provides structurally more stable inputs for SFE. This process compensates for the correlation information among electrode channels.

TAM is a lightweight temporal attention mechanism designed for the temporal characteristics of MI-EEG signals. Its core function is to guide the TCN to focus on time segments that are highly relevant to motor imagery tasks. Unlike the self-attention mechanism, which models global dependencies by computing the associations between temporal positions through Query-Key-Value operations, TAM only generates an independent attention score for each time step. The dependency modeling is then left to the subsequent TCN. The visualization experiments also demonstrate that TAM can guide temporal focusing without significantly increasing the number of parameters.

The experimental results in Section 3 demonstrate that the three modules described above cooperate from multiple dimensions to achieve a balance between model performance and computational efficiency.

However, this study still has several limitations that need further improvement. The improvement in cross-subject generalization remains limited, and the cross-domain representation capability of the model is still insufficient. Future work will attempt to introduce domain adaptation or transfer learning methods to further improve its generalization capability in cross-subject scenarios.

## 5 Conclusion

The practical deployment of BCI systems relies heavily on the accuracy and robustness of MI-EEG signal decoding algorithms. However, current mainstream methods still face several challenges. Multi-branch convolutional networks often fuse features of different scales through simple concatenation or element-wise addition, while neglecting the varying importance of features across branches, which may introduce redundant information. In addition, existing temporal feature modeling approaches struggle to simultaneously capture local transient responses and long-term rhythmic variations in MI-EEG signals, and they exhibit limited adaptability to inter-subject variability. To address these issues, this paper proposes a Weighted Multi-scale Attention-based Temporal Convolutional Network, which achieves efficient multi-scale feature fusion and refined spatiotemporal feature modeling through a modular design.

Experimental results on the BCI-2a and BCI-2b datasets demonstrate that WMA-TCNet achieves average accuracies of 85.8% and 90.0% in subject-dependent experiments, and 68.6% and 79.5% in cross-subject experiments, respectively, outperforming various existing mainstream methods.

At present, the evaluation of WMA-TCNet is mainly based on offline analysis, and it has not yet been validated in a real online BCI system. The stability and response latency of the model in real-time interactive scenarios still require further investigation. In future work, we will explore the scalability of WMA-TCNet under more complex task paradigms and cross-device conditions, aiming to facilitate its practical deployment and application in real-world BCI systems.

## Data Availability

The original contributions presented in the study are included in the article/Supplementary Material, further inquiries can be directed to the corresponding author.
