# Supplementary material for: A weighted multi-scale attention-enhanced temporal convolutional network for motor imagery EEG decoding in brain-computer interfaces
Source: Front Bioeng Biotechnol. 2026 Jun 3;14:1842610. doi: 10.3389/fbioe.2026.1842610 (PMC13272298; doi:10.3389/fbioe.2026.1842610)
Supplement: Supplementary file 1 [file Table1.docx]

Supplementary Material

# Supplementary Figures and Tables

TABLE 1 Detailed parameter settings for each module of the WMA-TCNet model

| Block | Layer | | Filters | Kernel size | Input | Output |
| --- | --- | --- | --- | --- | --- | --- |
| WMATCB | Conv2D | | $F1$ | (64×1) | ($T$, $Ch$, 1) | ($T$, $Ch$, $F1$) |
|  | Conv2D | | $F1$ | (32×1) | ($T$, $Ch$, 1) | ($T$, $Ch$, $F1$) |
|  | Conv2D | | $F1$ | (16×1) | ($T$, $Ch$, 1) | ($T$, $Ch$, $F1$) |
|  | GSA | |  |  | ($N$, $T$, $Ch$, 1) | ($T$, $Ch$, $F1$) |
|  | CP3 | | $F1$ | (1×1) | ($T$, $Ch$, 1) | ($T$, $Ch$, $F1$) |
| SFE | Depthwise Conv | | $F2$ | (1×$Ch$) | ($T$, $Ch$, $F1$) | ($T$, $1$, $F2$) |
|  | BN | |  |  | ($T$, $1$, $F2$) | ($T$, $1$, $F2$) |
|  | ELU | |  |  | ($T$, $1$, $F2$) | ($T$, $1$, $F2$) |
|  | AvgPooling | |  |  | ($T$, $1$, $F2$) | ($T/8$, $1$, $F2$) |
| STFF | Conv2D | | $F2$ | (16×1) | ($T/8$, $1$, $F2$) | ($T/8$, $1$, $F2$) |
|  | BN | |  |  | ($T/8$, $1$, $F2$) | ($T/8$, $1$, $F2$) |
|  | ELU | |  |  | ($T/8$, $1$, $F2$) | ($T/8$, $1$, $F2$) |
|  | AvgPooling | |  |  | ($T/8$, $1$, $F2$) | ($T/56$, $1$, $F2$) |
| OTS |  | |  |  | ($T/56$, $1$, $F2$) | ($T'$, $1$, $F2$) |
| TACNM | TAM | AvgPooling |  |  | ($T'$, $1$, $F2$) | ($T'$, $1$, $1$) |
|  |  | MaxPooling |  |  | ($T'$, $1$, $F2$) | ($T'$, $1$, $1$) |
|  |  | Concat |  |  |  | ($T'$, $1$, $2$) |
|  |  | Conv2D | 1 | (16×1) | ($T'$, $1$, $2$) | ($T'$, $1$, $1$) |
|  |  | Softmax |  |  | ($T'$, $1$, $1$) | ($T'$, $1$, $1$) |
|  |  | Output |  |  |  | ($T'$, $1$, $F2$) |
|  | TCN | Reshape |  |  | ($T'$, $1$, $F2$) | ($T'$, $F2$) |
|  |  | TCN Block |  |  | ($T'$, $F2$) | ($T'$, $32$) |
|  |  | TCN Block |  |  | ($T'$, $32$) | ($T'$, $32$) |
|  |  | Output |  |  | ($T'$, $32$) | (1, $32$) |
| FFO | Average | |  |  | ($n$, 1, $32$) | (1, $32$) |
|  | Dense | |  |  | (1, $32$) | $N_{class}$ |
|  | Softmax | |  |  | $N_{class}$ | $N_{class}$ |

TABLE 2 Dataset details

| **Datasets** | **Subjects** | **MI task** | $\boldsymbol{Ch}$ | **Time points** | **MI trails** |
| --- | --- | --- | --- | --- | --- |
| BCI-2a | 9 | Left hand、Right hand、foot、Tongue | 22 | 1125 | 5184 |
| BCI-2b | 9 | Left hand、Right hand | 3 | 1125 | 6480 |

TABLE 3 Ablation study of the WMA-TCNet model on the BCI-2a dataset and BCI-2b dataset

| Block | BCI-2a | | BCI-2b | |
| --- | --- | --- | --- | --- |
|  | Avg(%) | Kappa | Avg(%) | Kappa |
| WMA-TCNet | **85.8** | **0.807** | **90.0** | **0.796** |
| Without GSA | 82.5 | 0.764 | 89.6 | 0.791 |
| Without CP3 | 84.9 | 0.796 | 89.2 | 0.783 |
| Without OTS | 81.1 | 0.747 | 89.5 | 0.790 |
| Without TAM | 83.5 | 0.778 | 89.6 | 0.793 |
| Without TCN | 79.6 | 0.729 | 86.2 | 0.714 |

TABLE 4 Impact of different attention mechanisms on the performance of the WMA-TCNet model

| Attention mechanism | Parameters | Avg(%) | Kappa |
| --- | --- | --- | --- |
| Without Attention | 105.3k | 83.5 | 0.778 |
| With TAM | 105.5k | **85.8** | **0.807** |
| With MSA | 116.3k | 84.6 | 0.795 |

TABLE 5 Subject-dependent performance comparison results of the model on the BCI-2a dataset

| Methods | A01 | A02 | A03 | A04 | A05 | A06 | A07 | A08 | A09 | Avg(%) | Kappa |
| --- | --- | --- | --- | --- | --- | --- | --- | --- | --- | --- | --- |
| ShallowConvNet | 84.4 | **75.5** | 92.5 | 58.2 | 80.9 | 72.6 | 77.1 | **90.6** | 83.7 | 79.5* | 0.727 |
| DeepConvNet | 77.1 | 74.1 | 77.1 | 71.9 | 81.8 | 77.8 | 76.9 | 79.0 | 79.9 | 77.3* | 0.697 |
| EEGNet | 80.9 | 54.5 | 88.9 | 61.8 | 71.5 | 54.5 | 86.8 | 83.7 | 80.6 | 73.7** | 0.649 |
| MBEEG_SENet | 89.4 | 63.0 | 94.8 | 70.0 | 77.1 | 68.6 | 63.4 | 89.8 | 85.4 | 77.9* | 0.706 |
| ATCNet | 90.1 | 70.8 | **96.9** | 81.2 | 79.9 | 73.6 | 93.5 | 87.5 | 92.4 | 85.1 | 0.805 |
| EA-EEG | **90.7** | 65.0 | 96.7 | 81.2 | **83.0** | 60.9 | 94.2 | 85.3 | 83.7 | 82.3 | 0.762 |
| TFCA-TransNet | 88.2 | 64.4 | **96.9** | 82.6 | 77.1 | 71.5 | 94.5 | 90.1 | 86.8 | 83.6* | 0.779 |
| Proposed | 88.2 | 70.0 | 96.4 | **84.0** | 79.2 | **79.5** | **94.8** | 87.5 | **92.5** | **85.8** | **0.807** |

**Note：Bold highlights the best decoding accuracies. The * and ** indicate that the decoding accuracies of WMA-TCNet are signiﬁcantly higher than those of the compared methods with p< 0.05 and p< 0.01, respectively.**

TABLE 6 Subject-dependent performance comparison results of the model on the BCI-2b dataset

| Methods | B01 | B02 | B03 | B04 | B05 | B06 | B07 | B08 | B09 | Avg(%) | Kappa |
| --- | --- | --- | --- | --- | --- | --- | --- | --- | --- | --- | --- |
| ShallowConvNet | 72.5 | 65.4 | 79.4 | 97.5 | 92.8 | 90.0 | 86.9 | 92.8 | 85.9 | 84.8** | 0.696 |
| DeepConvNet | 71.6 | **73.6** | 87.2 | 96.6 | 93.4 | 84.1 | 87.8 | 92.2 | 81.9 | 85.4** | 0.707 |
| EEGNet | 77.2 | 69.3 | 88.4 | 98.8 | 98.1 | 90.6 | 93.1 | 94.7 | 91.3 | 89.1 | 0.781 |
| MBEEG_SENet | **81.3** | 70.0 | 86.9 | 98.4 | 97.8 | 90.3 | 91.9 | 95.3 | **92.2** | 89.3 | 0.787 |
| ATCNet | 80.8 | 71.9 | 88.8 | 98.4 | 97.8 | 90.5 | 94.7 | 94.7 | 87.0 | 89.4 | 0.793 |
| EA-EEG | 72.8 | 64.9 | 87.8 | 98.5 | 98.9 | 86.1 | **94.9** | 92.2 | 86.8 | 87.0* | 0.725 |
| TFCA-TransNet | **81.3** | 72.7 | 87.3 | **99.4** | **99.1** | 87.9 | 93.2 | 95.6 | 81.7 | 88.7 | 0.755 |
| Proposed | **81.3** | 72.4 | **89.9** | 98.4 | **99.1** | **91.6** | 93.1 | **96.3** | 88.2 | **90.0** | **0.796** |

**Note：Bold highlights the best decoding accuracies. The * and ** indicate that the decoding accuracies of WMA-TCNet are signiﬁcantly higher than those of the compared methods with p< 0.05 and p< 0.01, respectively.**

TABLE 7 Comparative analysis with existing methods

| Methods | Parameters | Inference time | FLOPs | Avg(%) | Kappa |
| --- | --- | --- | --- | --- | --- |
| ShallowConvNet | 46.1k | 37.98±0.93ms | 127.3M | 79.5 | 0.727 |
| DeepConvNet | 283.3k | 38.85±1.56ms | 227.1M | 77.3 | 0.697 |
| EEGNet | **2.6k** | **37.14±1.04ms** | **26.7M** | 73.7 | 0.649 |
| MBEEG_SENet | 10.5k | 38.26±1.03ms | 71.6M | 77.9 | 0.706 |
| ATCNet | 115.2k | 40.85±1.99ms | 82.3M | 85.1 | 0.805 |
| EA-EEG | 35.3k | 39.81±1.07ms | 55.1M | 82.3 | 0.762 |
| TFCA-TransNet | 253.3k | 41.38±1.99ms | 397.8M | 83.6 | 0.779 |
| Proposed | 105.5k | 40.13±1.16ms | 102.7M | **85.8** | **0.807** |

TABLE 8 Cross-subject performance comparison results of the model on the BCI-2a dataset and BCI-2b dataset

| Methods | BCI-2a | | BCI-2b | |
| --- | --- | --- | --- | --- |
|  | Avg(%) | Kappa | Avg(%) | Kappa |
| ShallowConvNet | 65.1 | 0.536 | 73.5 | 0.467 |
| DeepConvNet | 62.0 | 0.498 | 73.9 | 0.474 |
| EEGNet | 64.8 | 0.528 | 75.2 | 0.503 |
| MBEEG_SENet | 60.2 | 0.467 | 76.1 | 0.520 |
| TFCA-TransNet | 66.3 | 0.552 | 75.8 | 0.516 |
| Proposed | **68.6** | 0.581 | **79.5** | 0.590 |
